# Oncological outcomes of repeat metastasectomy for recurrence after hepatectomy for colorectal liver metastases. A case series

**DOI:** 10.1016/j.amsu.2020.01.006

**Published:** 2020-01-22

**Authors:** Yoshiaki Maeda, Toshiki Shinohara, Nozomi Minagawa, Ryota Koyama, Akihisa Nagatsu, Shingo Shimada, Tomonori Hamada

**Affiliations:** Department of Gastrointestinal Surgery, Hokkaido Cancer Center, Department of Surgery, Hokkaido Cancer Center, 4-2-3-54 Kikusui, Shiroishi, Sapporo, 003-0804, Japan

**Keywords:** Colorectal cancer, Liver metastases, Repeat resection, Metastasectomy, Hepatectomy

## Abstract

**Background:**

Although hepatectomy is the standard and only curative treatment for colorectal liver metastases, recurrence occurs in various organs, including the remnant liver, lung, peritoneum, and others. The outcomes and predictive factors of repeat metastasectomy for recurrence after initial hepatectomy remains controversial.

**Methods:**

We retrospectively assessed a consecutive series of 132 patients who underwent hepatectomy for colorectal liver metastases in a single institute.

**Results:**

There were 99 recurrence cases after initial hepatectomy, and 42 patients underwent metastasectomy (first repeat metastasectomy) to achieve R0 (17 liver cases, 16 lung cases, and 9 multiple or other cases), while 19 patients underwent subsequent second repeat metastasectomy (4 liver cases, 7 lung cases, and 8 multiple or other cases). Among the 99 recurrent cases after initial hepatectomy, the 5-year overall survival rate of the patients who underwent first repeat metastasectomy was significantly higher than that of chemotherapy/BSC (best supportive care) patients (60% vs. 14%, P < 0.0001). Furthermore, among the 26 recurrent cases after first repeat metastasectomy, the 5-year overall survival rate of the patients who underwent second repeat metastasectomy was significantly higher than that of chemotherapy/BSC patients (P = 0.024). A multivariate analysis revealed that lack of adjuvant chemotherapy, a short (<12 months) disease-free interval, and right-side colon primary were the independent poor prognostic factors for the overall survival after first repeat metastasectomy.

**Conclusion:**

The current study indicated that repeat metastasectomy for recurrence after initial hepatectomy for colorectal liver metastases could achieve a longer survival time, especially for patients with favorable predictive factors.

## Highlights

• Forty-two patients underwent repeat metastasectomy for recurrence after hepatectomy for colorectal liver metastases.
• Five-year survival rate of the repeat metastasectomy group was significantly higher than that of chemotherapy group.
• A short disease-free interval was the independent poor prognostic factors for survival after repeat metastasectomy.

## 1 Introduction

Colorectal cancer is the third-most common malignancy in the world, and approximately 50% of patients with colorectal cancer develop liver metastases at some time point in their disease course [1]. Liver resection is the standard and only curative treatment for colorectal liver metastases, and the 5-year survival rate after complete resection for patients with colorectal liver metastases is reported to be 40%–60% [[2], [3], [4]]. However, after initial hepatectomy, recurrence occurs in up to 70%–80% of the patients [5,6]. Although repeat hepatectomy has emerged as a viable therapy for liver limited recurrence [5,[7], [8], [9], [10]], the majority (50%–80%) of cases of recurrence involve extrahepatic organs, such as the lung and peritoneum [7,[10], [11], [12]]. Although chemotherapy including molecular-targeted agents for metastatic colorectal cancer has greatly improved over the last decade, the 5-year survival rate of patients with recurrent lesions treated with chemotherapy alone is less than 11% [5,[13], [14], [15], [16]].

The outcomes of repeat metastasectomy, including extrahepatic recurrence, after initial hepatectomy for colorectal metastases have been reported by some authors regarding lung and peritoneal metastasis [11,12,[17], [18], [19]]. However, the long-term survival benefit and prognosis factors for patients who undergo repeat surgery have yet to be determined.

This study aimed to clarify the oncological outcomes of repeat metastasectomy for recurrence to various organs after hepatectomy for colorectal liver metastases and to determine the prognostic factors after repeat surgery.

## 2 Patients and methods

A consecutive series of 132 patients who underwent hepatectomy (initial hepatectomy) for colorectal liver metastases in our institute treated from 2000 to 2016 were included in this study. We retrospectively assessed the characteristics of the patients and their survival after the treatments. This study was approved by ethical committee of the institute, and informed consent was obtained from the all presented patients. The study was registered with the Research Registry (researchregistry 5308) and has been reported in line with the PROCESS criteria [20].

Initial hepatectomy was performed if R0 resection was technically possible. From 2009, neo-adjuvant chemotherapy (six cycles of FOLFOX) was administered for cases with three or more liver nodules. Resectability was decided based on the size of the remnant liver volume (more than 30% functional liver remnant expected after the removal of all metastases), regardless of the number or the size of the liver metastases. Partial resection with a free margin of >1 cm was preferred, and anatomical resection was selected when needed. Even if patients had extra-hepatic metastases, both hepatectomy and extra-hepatic metastasectomy were performed when all lesions were resectable.

After initial hepatectomy, patients were routinely followed every three months by measuring the serum CEA and CA19-9 levels and performing contrast-enhanced computed tomography (CT) of the abdomen and thorax. Postoperative adjuvant chemotherapy was administered for some patients (mainly those with two or more metastases resected) depending on the surgeons' preference. Oxaliplatin-based regimens or oral UFT/LV were mainly used for adjuvant chemotherapy after initial hepatectomy. When relapse was diagnosed in the remnant liver, lung, peritoneum, or brain, repeat metastasectomy was performed if complete resection was possible. Metastasectomy for metastatic lesions was performed repeatedly as long as possible. All procedures were performed and supervised by staff surgeons certified by the Japan Surgical Society. When the recurrence lesions were diagnosed as unresectable, chemotherapy and/or best supportive care (BSC) were administered according to the patients’ status and will.

The overall and disease-free survival curves from the date of initial hepatectomy, first repeat metastasectomy, and second repeat metastasectomy were estimated using the Kaplan-Meier method. To investigate prognostic factors after surgery, the differences between the survival curves were analyzed using the log-rank test. Cox proportional hazards model among the variables was performed for the multivariate analysis. All statistical analyses were performed using the EZR statistical software program [21]. A value of *P* < 0.05 was considered to be statistically significant.

## 3 Results

**Fig. 1:**
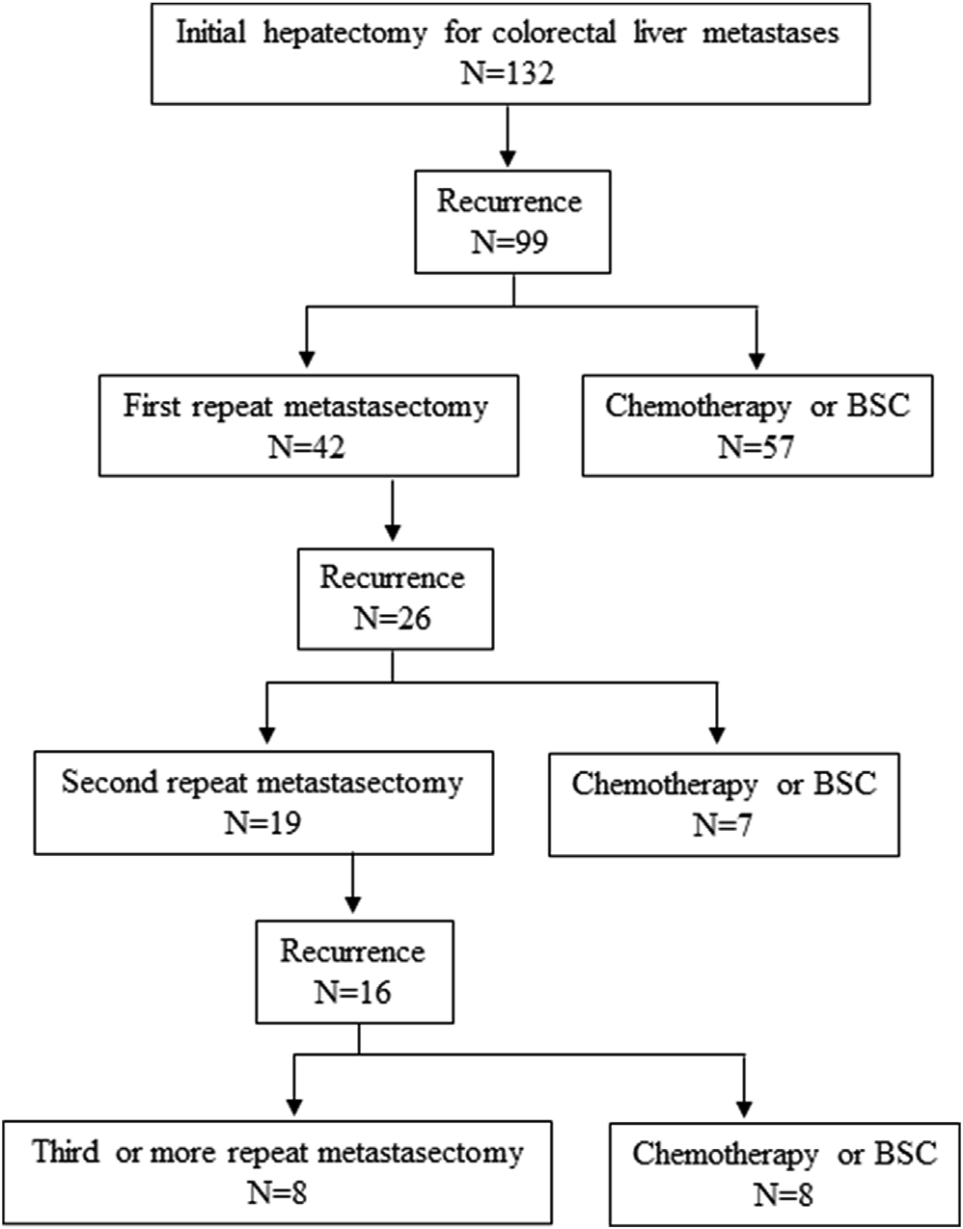
Flow diagram of the 132 patients and treatment procedures.

A total of 132 patients underwent initial hepatectomy for colorectal liver metastases between 2000 and 2016. A flow diagram of the 132 patients with initial hepatectomy is shown in Fig. 1. There were 99 recurrence cases after initial hepatectomy, and 42 of them underwent metastasectomy (first repeat metastasectomy) to achieve R0 (17 liver cases, 16 lung cases, and 9 multiple or other cases). The other 57 cases were diagnosed as unresectable and subsequently underwent chemotherapy and/or BSC. The reasons for unresectability were as follows: multiple liver and/or lung metastases in which R0 resection was considered impossible (27 cases); unresectable organ metastases, such as bone, brain, or distant lymph nodes (12 cases); carcinomatosa peritonitis (11 cases); and others (7 cases). After first repeat metastasectomy, there were 26 recurrence cases, and 19 of them underwent metastasectomy (second repeat metastasectomy) to achieve R0 again (4 liver cases, 7 lung cases, and 8 multiple or other cases).

The clinical characteristics of the patients at initial hepatectomy, first repeat metastasectomy, and second repeat metastasectomy are summarized in Table 1. Adjuvant chemotherapy was administered for 68% (90/132) of the patients after initial hepatectomy, 60% (25/42) of the patients after first repeat metastasectomy, and 58% (11/19) of the patients after second repeat metastasectomy. There was one 30-day mortality after initial hepatectomy (1/132, 0.8%), whereas no surgical mortalities were observed after first and second repeat metastasectomy.

**Table 1:** Characteristics of patients underwent Initial hepatectomy, first and second repeat metastasectomy for metastases from colorectal cancer.

Variable	Patients number		
	Initial hepatectomy	First repeat metstasectomy	Second repeat metstasectomy
	(N = 132)	(N = 42)	(N = 19)
Age (<70 years old/70<)	100/32	37/5	18/1
Gender (Female/Male)	45/87	15/27	6/13
Depth of primary tumor (T1-T3/T4)	100/32	29/13	14/5
Lymph node metastases of primary (N0/N+)	49/83	14/28	7/12
Primary site (Rt colon/Lt colon/Rectum)	33/59/40	11/26/11	4/10/5
Number of metastases at initial hepatectomy (one/two or more)	68/64	19/23	9/10
Maximum size of at initial hepatectomy (<5 cm/5 cm<)	115/17	35/7	16/3
Neoadjuvant chemotherapy before initial hepatectomy (Yes/No)	38/94	14/28	6/13
Adjuvant chemotherapy after initial hepatectomy (Yes/No)	90/42	31/11	11/8
Adjuvant chemotherapy after repeat metastasectomy (Yes/No)	–	25/17	11/8
DFI (primary to initial hepatectomy) (<12 m/12 m<)	98/34	34/8	16/3
DFI (initial hepatectomy to first repeat) (<12 m/12 m<)	–	26/16	12/7
DFI (First to second repeat metstasectomy) (<12 m/12 m<)	–	–	10/9
Extra-hepatic metastases at initial hepatectomy (Present/No)	22/110	10/32	5/10
CEA level at initial hepatectomy (<20 ng/ml/20 ng/ml<)	82/50	24/18	13/6
Number of metastases at repeat surgery (one/two or more)	–	22/20	11/8
Sites of metastasectomy (Liver/Lung/Others^a^)	132/0/0	17/16/9	4/7/8
30-days surgical mortality	0.8% (1/132)	0%	0%

a Liver and lung: n = 3, lung and peritoneum: n = 2, ovary: n = 2, liver and peritoneum: n = 1, and lymph node: n = 1.

**Fig. 2:**
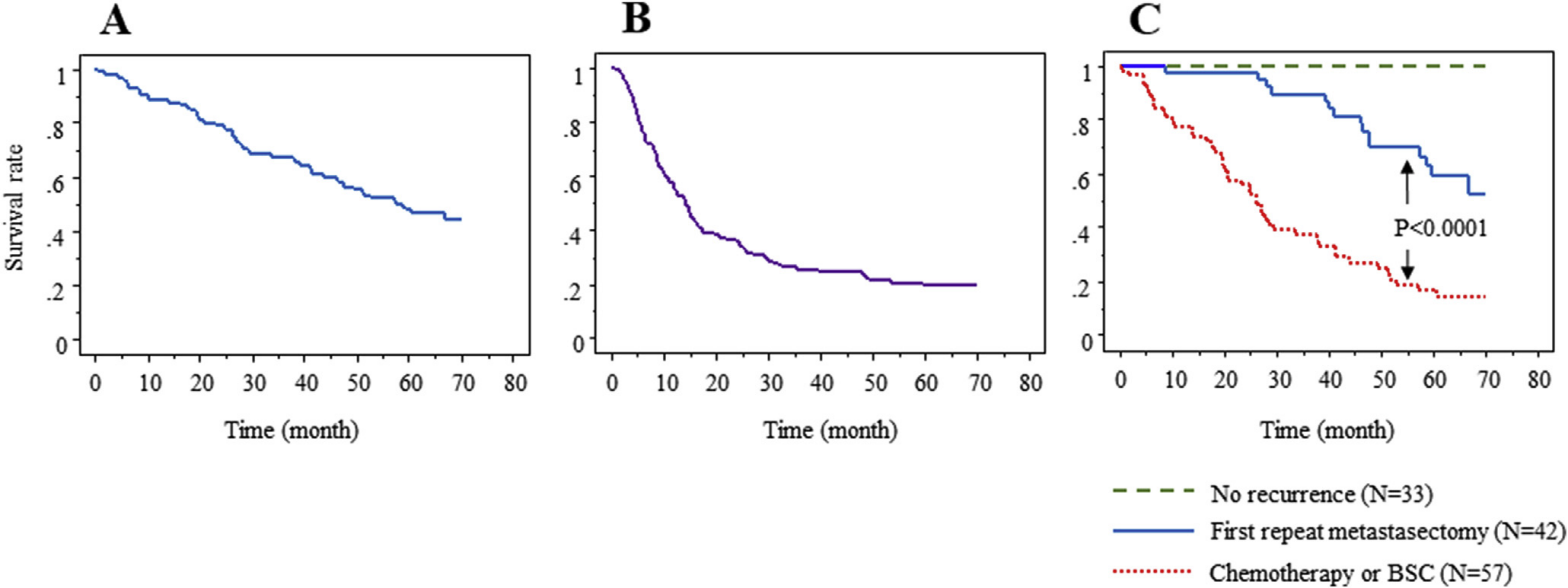
Kaplan-Meier analysis of the overall (A) and disease-free (B) survival after initial hepatectomy for colorectal liver metastases. (C) The overall survival curves of patients with no recurrence, first repeat metastasectomy, and chemotherapy/BSC. The overall survival rate of the patients who underwent first repeat metastasectomy was significantly higher than that of the chemotherapy/BSC patients (P < 0.0001). The predicted 5-year survival rate in the first repeat metastasectomy patients was 60%.

The 5-year overall and disease-free survival rates after initial hepatectomy were 49% and 21%, respectively (Fig. 2A and B). Among the recurrent patients after initial hepatectomy, the 5-year overall survival rates of the patients who did and did not undergo first repeat metastasectomy were 60% and 14%, respectively. The 5-year survival rate of the patients who underwent first repeat metastasectomy was significantly (P < 0.0001) higher than that of the chemotherapy/BSC patients (Fig. 2C).

**Fig. 3:**
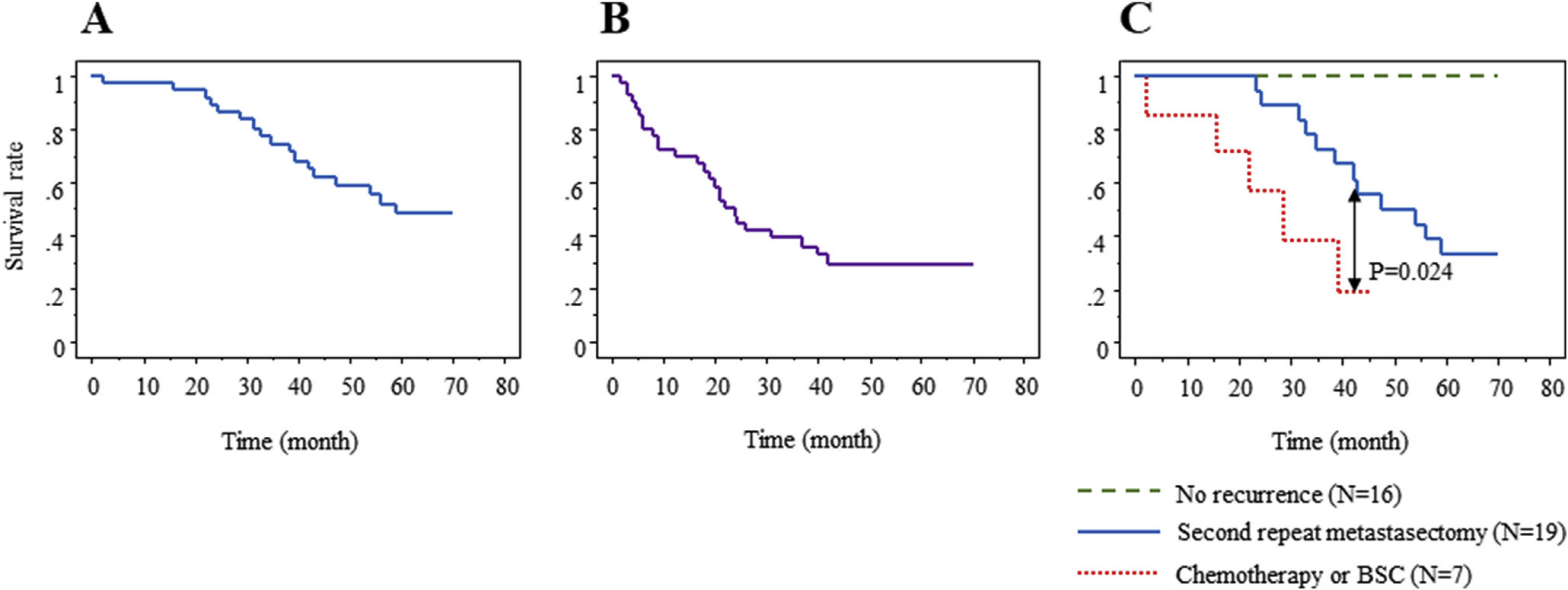
Kaplan-Meier analysis of the overall (A) and disease-free (B) survival after first repeat metastasectomy following initial hepatectomy. (C) The overall survival curves of patients with no recurrence, second repeat metastasectomy, and chemotherapy/BSC. The overall survival of the patients who underwent second repeat metastasectomy was significantly higher than that of the chemotherapy/BSC patients (P = 0.024). The predicted 5-year survival rate in the second repeat metastasectomy patients was 33%.

The 5-year overall and disease-free survival rates after first repeat metastasectomy were 48% and 30%, respectively (Fig. 3A and B). Among recurrent patients after first repeat metastasectomy, the 5-year overall survival rate of the patients who underwent second repeat metastasectomy was 33%. There were no 5-year survivors among the chemotherapy/BSC patients at the latest follow-up. The survival rate of the patients who underwent second repeat metastasectomy was significantly (P = 0.024) higher than that of the chemotherapy/BSC patients (Fig. 3C).

The potential prognostic factors predicting the overall and disease-free survival after first metastasectomy are shown in Table 2. Right-side colon primary (P = 0.023), lack of adjuvant chemotherapy following initial hepatectomy (P = 0.028), a short (<12 months) disease-free interval between initial hepatectomy and first repeat metastasectomy (P = 0.0057), and extra-hepatic metastases at initial hepatectomy (P = 0.042) were identified as significant poor prognostic predicters for the overall survival after first repeat metastasectomy. The sites of resection (liver-limited, lung-limited, or others) were not a significant prognostic factor after first repeat metastasectomy (Table 2, left column). Lymph-node metastases of primary tumor (P = 0.012) and a short (<12 months) disease-free interval (P = 0.011) were identified as significant negative predictive factors for the disease-free survival (Table 2, right column).

**Table 2:** Factors associated with overall and disease-free survival on univariate analysis for patients who underwent first repeat metastasectomy after hepatectomy for CLM.

Variable	Overall survival			Disease-free survival		
	5-y OS	Median OS	P	5-y DFS	Median DFS	P
	(%)	(month)		(%)	(month)	
Age						
70 or <70 years old	49	59	0.36	35	23	0.48
70 years old<	50	60<		NA	37	
Gender						
Female	73	60<	0.11	24	18	0.35
Male	38	53		31	31	
Depth of primary tumor						
T1-T3	60	60<	0.054	37	31	0.053
T4	15	47		NA	9	
Lymph node metastases of primary tumor						
N0	34	38	0.24	NA	17	0.012
N1<	53	60<		38	31	
Right side primary (Cecum-Transverse colon)						
Right	27	34	0.023	20	9	0.057
Left-rectum	54	60<		31	26	
Chemotherapy before initial hepatectomy						
Yes	63	60<	0.36	17	22	0.40
No	43	53		35	24	
Chemotherapy after initial hepatectomy						
Yes	60	60<	0.028	30	26	0.59
No	16	43		30	21	
Chemotherapy after repeat metastasectomy						
Yes	59	60<	0.26	27	22	0.72
No	37	53		32	26	
DFI (Primary resection to initial hepatectomy)						
<12 months	49	56	0.67	28	22	0.29
12 months<	51	60<		36	37	
DFI (Initial hepatectomy to repeat metstasectomy)						
<12 months	28	46	.0057	11	20	0.011
12 months<	77	60<		58	60<	
Extra-hepatic metastases at initial hepatectomy						
Present	20	43	0.042	0	19	0.13
No	56	60<		36	26	
CEA level at initial hepatectomy						
<20 ng/ml	52	60<	0.67	18	22	0.23
20 ng/ml<	43	54		41	40	
Nunmer of metastases at repaet metastasectomy						
1	53	60<	0.36	33	31	0.73
2 or more	42	56		22	22	
Type of metastasectomy						
Hepatectomy only	52	60<	0.17	38	31	0.37
Lung resection only	34	43		26	15	
Others	72	60<		20	26	

OS overall survival, DFS disease free survival, NA not assessed.

A multivariate analysis revealed that a lack of adjuvant chemotherapy following initial hepatectomy (HR 5.41), a short (<12 months) disease-free interval between initial hepatectomy and first repeat metastasectomy (HR 12.9), and right-side colon primary (HR 4.26) were independent poor prognostic factors for the overall survival after first repeat metastasectomy (Table 3, left column). Regarding the disease-free survival, a short (<12 months) disease-free interval (HR 3.26), and lymph-node metastases of the primary tumor (HR 2.95) were the independent poor prognostic factors (Table 3, right column). A short (<12 months) disease-free interval between initial hepatectomy and first repeat metastasectomy was the only poor prognostic factor for the both overall and disease-free survival after first repeat metastasectomy.

**Table 3:** Multivariate analysis for factors associated with overall and disease-free survival of patients who underwent first repeat metastasectomy after initial hepatectomy for CLM.

Variable	Overall survival			Disease-free survival		
	HR	95% CI	P value	HR	95% CI	P value
Adjuvant chemotherapy after initial hepatectomy	5.41	1.49–19.6	0.010	1.16	0.44–3.03	0.77
DFI (initial hepatectomy to first repeat metstasectomy)	12.9	2.32–71.7	0.0035	3.26	1.24–8.52	0.016
Extra-hepatic metastases at initial hepatectomy	1.04	0.26–4.22	0.95	1.13	0.40–3.23	0.82
Right side primary (Cecum-Transverse colon)	4.26	1.08–16.8	0.039	2.13	0.83–5.51	0.12
Lymph node metastases of primary tumor	1.34	0.41–4.39	0.63	2.95	1.03–8.47	0.045
T4 lesion	2.57	0.85–7.75	0.094	1.84	0.77–4.39	0.17

HR hazard ratio, CI confidence interval, DFI disease free.

## 4 Discussion

Hepatectomy has been widely accepted as the standard and only curative treatment for colorectal liver metastases, and the 5-year survival rate after complete resection of liver metastases is reported to be 40%–60% [[2], [3], [4]]. However, recurrence occurs in up to 70%–80% of patients after initial hepatectomy [5,6,22]. Recently, many authors have reported the efficacy and safety of repeat hepatectomy for liver-limited recurrence after initial hepatectomy for colorectal liver metastasis, with 5-year survival rates of 20%–70% [5,[7], [8], [9], [10]]. Although repeat hepatectomy has been accepted as an excellent treatment option for liver-limited recurrence, the majority (50–80%) of recurrence cases involve extrahepatic organs, such as the lung and peritoneum [7,[10], [11], [12],^23^]. As a result, the percentage of patient benefited by repeat hepatectomy is relatively low (6–32%) [[24], [25], [26]]. Given that the majority of recurrences after hepatectomy for colorectal liver metastases develop outside the liver, strategies including resection for extra-hepatic metastases are important to consider in order to improve the survival rate after initial hepatectomy. Some studies have shown that complete resection of entire metastases, including extrahepatic lesions, can achieve favorable long-term outcomes [27,28]. The difference in the survival between patients with and without extrahepatic disease was not shown to be significant when complete R0 resection was achieved [29,30]. However, many studies have reported that extra-hepatic recurrence is associated with a poor prognosis among recurrent cases after initial hepatectomy [26,31,32]. At present, few studies described the outcomes of repeat metastasectomy for various organs including not only the liver but also the lung, peritoneum, and other organs after initial hepatectomy [11,12,[17], [18], [19]]. Furthermore, the prognostic factors for repeat metastasectomy have not yet been described.

The current study clearly demonstrated the survival benefit of both first and second repeat metastasectomy for recurrence after initial hepatectomy for colorectal liver metastases. In the present study, 43% of the patients with recurrence after initial hepatectomy underwent first repeat metastasectomy. While these were a highly select group of patients, the survival rate of the patients who underwent repeat metastasectomy was significantly higher than that of the chemotherapy/BSC patients. Furthermore, approximately 70% of the patients with recurrence after first repeat metastasectomy underwent second repeat metastasectomy, and their survival rate was also significantly higher than that of chemotherapy/BSC patients. The 5-year overall survival rates after first and second metastasectomy were similar to those in previous reports on repeat metastasectomy including extra-hepatic recurrence following initial hepatectomy [11,17]. Although chemotherapy including molecular-targeted agents for metastatic colorectal cancer has greatly improved over the last decade, the reported 5-year survival rates of patients with metastatic or recurrent colorectal cancer treated with chemotherapy alone are less than 11% [[13], [14], [15], [16]]. The survival rates after first and second metastasectomy in the current study were markedly higher than the reported results of systemic chemotherapy.

Another novel point of this report is the identification of prognostic factors associated with both the overall and disease-free survival after repeat metastasectomy for recurrence cases including extra-hepatic metastases. According to a multivariate analysis, the following three factors were poor prognostic indicators for the overall survival: a lack of adjuvant chemotherapy after initial hepatectomy, a short (<12 months) disease-free interval between initial hepatectomy and first repeat metastasectomy, and right-side colon primary lesion. Regarding the disease-free survival, a short disease-free interval as well as lymph-node metastases of the primary tumor were the independent poor prognostic factors. A short (<12 months) disease-free interval between initial hepatectomy and first repeat metastasectomy was the only poor prognostic factor for both the overall and disease-free survival after first repeat metastasectomy, and strongly predicted the prognosis of the patients after first repeat metastasectomy. Studies to date have yielded conflicting results regarding the prognostic factors after repeat metastasectomy including liver and extra-hepatic recurrence. However, no other studies but Yang's have clearly identified the prognostic factors after repeat metastasectomy including extra-hepatic metastases. In their report, a short disease-free interval was also identified as a poor prognostic factor, along with R1 resection [18]. Of note, a short disease-free interval has also been identified as a negative prognostic factor in many reports regarding repeat hepatectomy for liver-limited recurrence following initial hepatectomy [7,28,33,34].

In the present study, the administration of adjuvant chemotherapy was found to be a positive prognostic factor after first metastasectomy, although only 68% of the patients received adjuvant chemotherapy. The administration of adjuvant chemotherapy was left to the choice of the surgeon in this study. The main reason for the low rate of performing chemotherapy was the lack of definite evidence concerning the benefit of adjuvant chemotherapy. The survival benefit of adjuvant chemotherapy after R0 initial hepatectomy for colorectal liver metastases is controversial at present [35]. However, several recent studies have suggested that select patients, especially those with synchronous liver metastases are favorably indicated for adjuvant chemotherapy after initial hepatectomy [36].

The primary cancer sit (right-side colon) was also identified as a poor prognostic factor after first metastasectomy in the present study. Many authors have reported that a right-sided primary tumor was associated with a poor survival after surgery for colorectal liver metastases [37]. The relatively aggressive biological nature of right-side colon cancer, showing an increased incidence of RAS mutations may be the main reason for this [38].

In the present study, the target organ of the first metastasectomy (liver, lung, or others) did not significantly affect the survival rate. The 5-year overall survival rate after pulmonary resection as the first metastasectomy was 34%, which was compatible with the reported data for repeat pulmonary resection for colorectal lung metastases [39,40]. Although some authors claim that prior hepatectomy for liver metastasis is a significantly poor prognostic factor after surgery for lung metastasis [41], many other studies have shown that there are no significant differences in the overall survival between liver and lung metastases cases when complete repeat metastasectomy is achieved [18,40]. These data support the surgical approach for not only intra-hepatic but also extra-hepatic recurrence after initial hepatectomy if complete resection is technically possible.

The present findings suggest that the benefit of resections of metastatic colorectal cancer was maintained consistently across initial hepatectomy, first repeat metastasectomy, and second repeat metastasectomy. Although the overall survival rates of the patients who underwent first and second repeat metastasectomy were good enough to make this approach acceptable for treatment selection, the recurrence rates were relatively high. The recurrence rate has been reported to be significantly higher after repeat surgery than after initial hepatectomy [42]. Given the high recurrence rates after repeat metastasectomy, many authors have proposed a strategy involving performing neoadjuvant chemotherapy before repeat metastasectomy, especially for patients with poor prognostic factors [7,22,27,[43], [44], [45]]. Their claim is that neoadjuvant chemotherapy has potential utility for evaluating the responsiveness to chemotherapy and detecting further metastases in the interim period prior to repeat resection. They also have emphasized the role of neoadjuvant chemotherapy in selecting patients most suited for surgery. Figueras et al. reported that adjuvant chemotherapy significantly improved the survival after liver resection for the patients with high-risk factors [46]. Based on the results of the current study, strategies including both repeat metastasectomy and perioperative chemotherapy should be recommended for resectable recurrence cases with poor prognostic factors (a lack of adjuvant chemotherapy following initial hepatectomy, a short disease-free interval, and right-side colon primary cancer).

Several limitations associated with the present study warrant mention. For example, this study is a retrospective analysis of a highly selected patient group. A prospective randomized study will therefore be needed to clearly demonstrate the survival benefits of repeat surgery for recurrence after initial hepatectomy for colorectal liver metastases, although this is very difficult and ethically controversial.

## 5 Conclusion

The current study indicated that repeat metastasectomy for recurrence after initial hepatectomy for colorectal liver metastases could achieve a longer survival time, especially for patients with favorable predictive factors.

### Statement of ethics

The authors have no ethical conflicts to disclosure. This study was approved by ethical committee of the institute, and informed consent was obtained from the all presented patients. The study was registered with the Research Registry (researchregistry 5308) and has been reported in line with the PROCESS criteria [20].

## Funding

This study was not supported by any grant.

## Provenance and peer review

Not commissioned, externally peer reviewed.

## Ethical approval

This study was approved by the ethical committee of the hospital (Approved No. 31–21).

## Author contribution

Study conception and design: Maeda, Shinohara.

Acquisition of data: Maeda, Shinohara, Koyama, Minagawa.

Operator of surgery: Maeda, Shinohara, Nagatsu, Hamada.

Drafting of manuscript: Maeda, Shimada, Minagawa.

## Consent

Written informed consent was obtained from the all presented patients.

## Registration of research studies

The study was registered with the Research Registry (researchregistry 5308).

## Guarantor

Yoshiaki Maeda, M.D., PhD, Department of Gastrointestinal Surgery, Hokkaido Cancer Center, Mail address: Department of Surgery, Hokkaido Cancer Center, 4-2-3-54 Kikusui, Shiroishi, Sapporo, 003–0804 JAPAN, E-mail address: maeda 19671101@yahoo.co.jp, Telephone: +81-11-811-9111, Fax: +81-11-832-0652.

## Declaration of competing interest

The authors have no financial interests or potential conflicts of interest.
